# Formulation Development, Evaluation and Comparative Study of Effects of Super Disintegrants in Cefixime Oral Disintegrating Tablets

**DOI:** 10.4103/0975-1483.66794

**Published:** 2010

**Authors:** KS Remya, P Beena, PV Bijesh, A Sheeba

**Affiliations:** *Nazareth College of Pharmacy, Othera P.O, Thiruvalla, Pathanamthitta (Dist.), Kerala - 689 546, India*

**Keywords:** Cefixime, superdisintegrants, oral disintegrating tablets

## Abstract

The present work was aimed at formulation development, evaluation and comparative study of the effects of superdisintegrants in Cefixime 50 mg oral disintegrating tablets. The superdisintegrants used for the present study were sodium starch glycolate and crosscarmellose sodium. The formulated tablets were evaluated for various tableting properties, like hardness, thickness, friability, weight variation, disintegration time and dissolution rate. Comparative evaluation of the above-mentioned parameters established the superiority of the tablets formulated with crosscarmellose sodium to those formulated with sodium starch glycolate.

## INTRODUCTION

Disintegrants are agents added to tablet formulations to promote the break-up of the tablet into smaller fragments in an aqueous environment, thereby increasing the available surface area and promoting a more rapid release of the drug substance. In more recent years, several newer disintegrants have been developed, often called “super disintegrants.” These newer substances can be used at lower levels than conventionally used disintegrants. Three major mechanisms and factors affecting tablet disintegration are suggested as swelling, porosity and capillary action and deformation. Three major group of compounds that have been developed as superdisintegrants are modified starches, cross-linked polyvinylpyrrolidone and modified cellulose.

One of the major streams of application of superdisintegrants is in the formulation of oral disintegrating tablets/mouth dissolving tablets. An oral disintegrating tablet is a solid dosage form that disintegrates and dissolves in the mouth, either on or beneath the tongue or in the buccal cavity without water, within 60 s or lower. The US Food and Drug Administration (FDA) Center for Drug Evaluation and Research (CDER) defines, in the orange book, an oral disintegrating tablet as, “*A solid dosage form containing medicinal substances, which disintegrates rapidly, usually within a matter of seconds, when placed upon the tongue.*” At present, oral disintegrating tablets are the only quick-dissolving dosage form recognized by FDA and listed in the approved drug products with therapeutic equivalence evaluations.[1,2]

The drug selected for the study was cefexime trihydrate, which is used in the treatment of uncomplicated urinary tract infections.[3,4] The aim of the study was to formulate an oral disintegrating tablet of cefixime trihydrate using two superdisintegrants separately (crosscarmellose sodium and sodium starch glycolate), and to select the best among the two based on the disintegration time and other tableting properties.

## MATERIALS AND METHODS

### Materials

Cefixime trihydrate was procured from Aurobindo Pharma Ltd. Sodium starch glycolate and croscarmellose sodium were procured from DK Enterprises, while magnesium stearate and talc was from Nice Chemicals.

### Methodology

#### Preformulation studies[5–7]

Preformulation study is defined as an investigation of the physical and chemical properties of drug substance alone and when combined with the excipients. The overall objective of preformulation testing is to generate information useful to the formulator in developing a stable and bioavailable dosage form that can be mass produced. The commonly investigated preformulation parameters include angle of repose, bulk density/tapped density, pour density, Carr’s compressibility index and Hausner ratio.

#### Angle of repose

It is determined by allowing a powder to flow through a funnel and fall freely on to a surface. Further addition of powder is stopped as soon as the pile touches the tip of the funnel. A circle is drawn around the pile without disturbing it. The height and diameter of the resulting cone are measured. The same procedure is repeated three times and the average value is taken. Angle of repose is calculated by using the following equation:

Tan θ = h/r

Where, h = height of the powder cone; r = radius of the powder

### Bulk density

Unless otherwise specified, pass a quantity of material sufficient to complete the test through a 1.00-mm (no. 18) screen to break up agglomerates that may have formed during storage. Into a dry 250-ml cylinder introduce, without compacting, approximately 100 g of the test sample (M) weighed with 0.1% accuracy. If it is not possible to use 100 g, the amount of the test sample and the volume of the cylinder may be modified. Select a sample mass having an untapped apparent volume of 150–250 ml. A 100-ml cylinder is used for apparent volumes between 50 and 100 ml. Fill the cylinder carefully. Carefully level the powder without compacting, if necessary, and read the unsettled apparent volume (Vo). Calculate the bulk density, in g/ml, using the formula,

Bulk density = M/Vo

### Tapped density

Accurately weighed quantity of powder is introduced into a measuring cylinder. Mechanically tap the cylinder containing the sample by raising the cylinder and allowing it to drop under its own weight using a suitable mechanical tapped density tester at a nominal rate of 300 drops/min. Tap the cylinder 500 times and measure the tapped volume (Va). Repeat the operation for an additional 750 tappings and again measure the tapped volume as (Vb).

If the difference between Va and Vb is <2%, Vb is the final tapped volume (Vf). If the difference is higher, repeat the tapings for an additional 1,250 times, and then the tapped density can be calculated using the following formula (United States pharmacopoeia, 2004)

Tapped density = M/Vf

Where, M = weight of the sample taken; Vf = final tapped volume

### Carr’s index

The compressibility index of granules can be determined using Carr’s compressibility index, and can be determined by the following formula:

$$\mathrm{Carr}’s\;\mathrm{index}\;\left(\%\right)\;=\;\frac{\left(\mathrm{Tapped}\;\mathrm{density}\;-\;\mathrm{Pour}\;\mathrm{density}\right)}{\mathrm{Tapped}\;\mathrm{density}}\;\times \;100$$

### Hausner ratio

The Hausner ratio can be determined using the following formula:

$$\mathrm{Hausner}\;\mathrm{ratio}\;\left(\%\right)\;=\;\frac{\mathrm{Tapped}\;\mathrm{density}}{\mathrm{Pour}\;\mathrm{density}}\;\times \;100$$

### Compatibility studies

IR studies of drug and drug with superdisintegrants were carried out in order to check the compatibility between the drug and the excipients.

### Formulation development

The methodology selected for the preparation of cefixime oral disintegrating tablets is direct compression. About 10 formulations were prepared, of which five formulations included varying concentrations of the superdisintegrant sodium starch glycolate and five of crosscarmellose sodium. The list of ingredients is given in Tables 1 and 2.

**Table 1:** Formulation of cefixime with sodium starch glycolate

Ingredients	S _1(mg)_	S_2(mg)_	S_3(mg)_	S_4(mg)_	S_5(mg)_
Cefixime trihydrate	55.90	55.90	55.90	55.90	55.90
Microcrystalline cellulose	214.30	211.50	-	-	217.10
Pregelatinised starch	-	-	214.30	211.50	-
Sodium starch glycolate	5.60	8.40	5.60	8.40	2.80
Magnesium stearate	1.40	1.40	1.40	1.40	1.40
Talc	2.80	2.80	2.80	2.80	2.80
Average weight (mg)	280.00	280.00	280.00	280.00	280.00

**Table 2:** Formulation of cefixime with cross carmellose sodium

Ingredients	C_1(mg)_	C_2(mg)_	C_3(mg)_	C_4(mg)_	C_5(mg)_
Cefixime trihydrate	55.90	55.90	55.90	55.90	55.90
Microcrystalline cellulose	214.30	211.50	-	-	217.10
Pregelatinised starch	-	-	214.30	211.50	-
Sodium starch glycolate	5.60	8.40	5.60	8.40	2.80
Magnesium stearate	1.40	1.40	1.40	1.40	1.40
Talc	2.80	2.80	2.80	2.80	2.80
Average weight (mg)	280.00	280.00	280.00	280.00	280.00

### Tablet evaluation[8–10]

The selected batches made in bulk were subjected to evaluations as per Indian pharmacopoeia.

### Weight variation

Twenty tablets were selected at random, weighed and the average weight was calculate. Not more than two of the individual weights should deviate from the average weight by more than 5%.

### Friability

For each formulations, preweighed tablet samples (20 tablets) were placed on the friabilator, which is then operated for 100 revolutions. The tablets were then dusted and reweighed. Conventional compressed tablets that loose <0.5–1.0% of their weight are considered acceptable.

### Hardness

Tablet hardness of each formulation was determined using a Monsanto hardness tester. Results were calculated from the average results of six tablets.

### Thickness

Tablet thickness is determined using vernier calipers. Six tablets were evaluated to determine the average thickness.

### Disintegration test

Introduce one tablet into each tube and add a disc to each tube. Suspend the assembly in the beaker containing the specified liquid and operate the apparatus for a specified period of time. The tablet passes the test if all tablets have disintegrated. If one or two tablets fail to disintegrate, repeat the test on 12 additional tablets, such that not <16 of the total of 18 tablets tested disintegrate. If the tablets adhere to the disc, repeat the test by omitting the disc. The preparation complies with the test if all the tablets in the repeat test disintegrate.

### Dissolution studies/*in vitro* release studies

#### Medium

0.05 M potassium phosphate buffer, pH 7.2, prepared by dissolving 6.8 g of monobasic potassium phosphate in 1,000 ml of water and adjusting with 1N sodium hydroxide to a pH of 7.2, 900 ml.

#### Apparatus

Dissolution apparatus with 100 rpm.

#### Time

Forty-five minutes.

#### Procedure

The amount of cefixime released was determined by measuring the absorbance of the sample withdrawn at 280 nm in comparison with a standard solution having a known concentration of USP cefixime reference standard (RS) in the same medium.

## RESULTS AND DISCUSSION

### Preformulation studies

Values of the preformulation studies are given in Table 3, from which it is evident that all the parameters analyzed showed satisfactory flow properties and compression characteristics.

**Table 3:** Physicochemical evaluation of the formulations

Parameters	Formulation with cross carmellose mean value of (C1 – C5)	Formulation with sodium starch glycollate (S1 – S5)
Angle of repose	24 ° 37’ 2.1”	27° 25’ 13.02”
Bulk density / tapped density	0.6517	0.7117
Pour density	0.5140	0.5761
Carr’s compressibility index	21.12	19.05
Hausner ratio	1.26	1.23

### Compatibility studies

**Figure 1:**
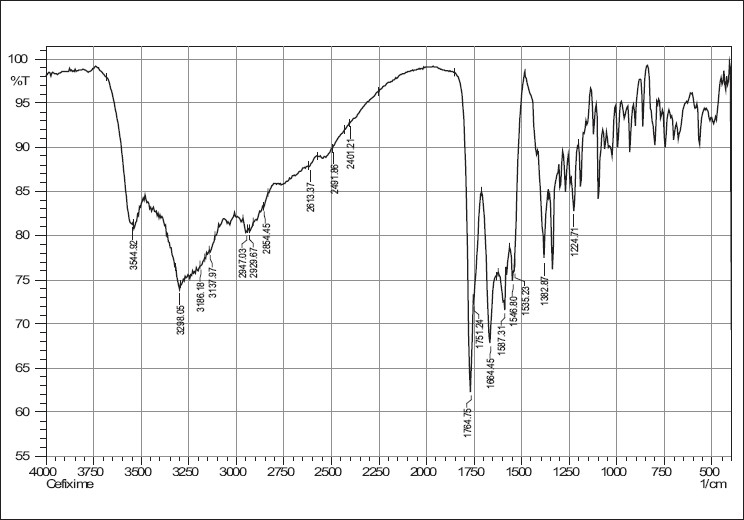
IR spectrum of cefixime

**Figure 2:**
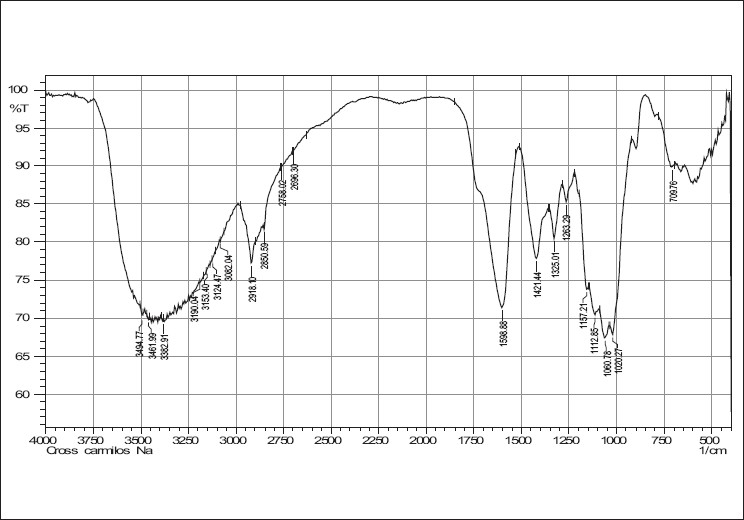
IR spectrum of crosscarmellose sodium

**Figure 3:**
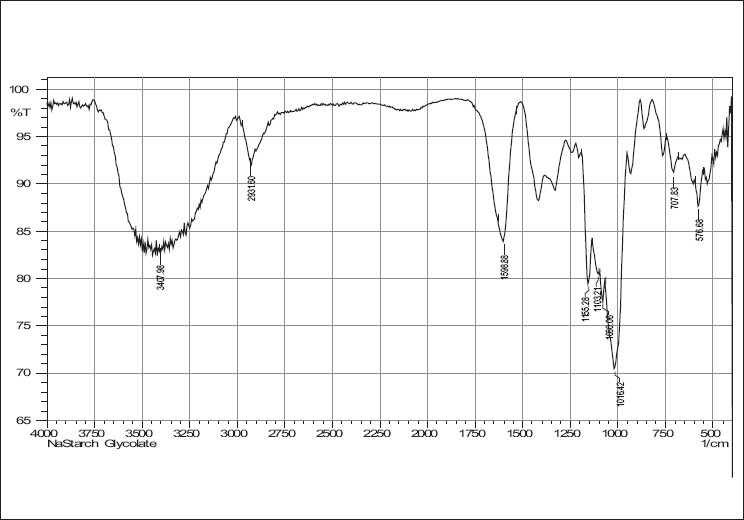
IR spectrum of sodium starch glycollate

**Figure 4:**
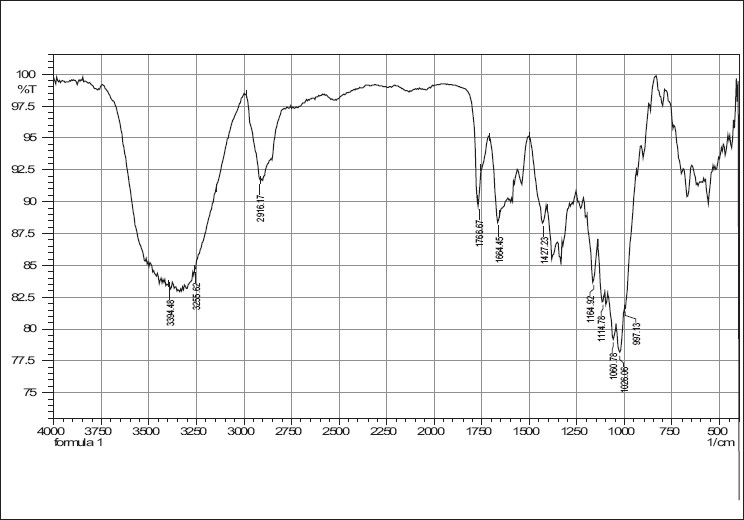
IR spectrum of cefixime tablet blend with crosscarmellose sodium

**Figure 5:**
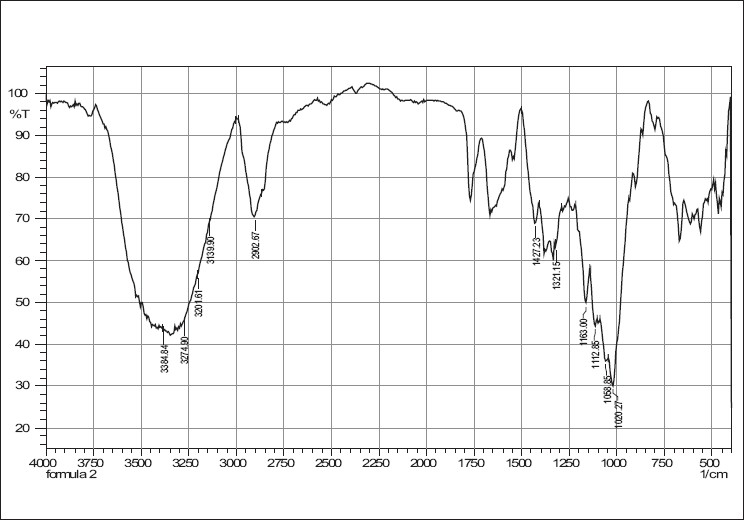
IR spectrum of cefixime tablet blend with sodium starch glycolate

IR spectra of cefixime, sodium starch glycolate, crosscarmellose sodium and combinations are given in Figures 1–5. The results of the FTIR spectral analysis showed that the peaks and the pattern of the spectra were similar in all cases, which indicated that there was no chemical interaction or decomposition of cefixime during the preparation of the tablets.

### Formulation

Because the aim of the project was to formulate cefixime 50 mg DT tablet, which disintegrated within 1 min, priority was given so as to get good results with cost-effectiveness of the product.

From the tables and histograms, it was evident that the formulations C1 and S3 stood ahead in all the tableting properties and in disintegration performance. Both the batches were prepared in bulk [Table 4] and confirmatory evaluation tests were carried out.

**Table 4:** Tablet ingredients for scale up batch

Ingredients	Quantity/ tablet (mg)	Quantity for 50 tablets (g) (S_3_)	Quantity for 50 tablets (g) (C _1_)
Cefixime trihydrate	55.90	2.795	2.795
Microcrystalline cellulose	214.30	10.715	10.715
Sodium starch glycolate	5.60	0.280	----
Cross carmellose sodium	5.60	----	0.280
Magnesium stearate	1.40	0.070	0.070
Talc	2.80	0.140	0.140
Pineapple flavor	q.s	q.s	q.s
Tarazarine lake	q.s	q.s	q.s
Average weight (mg)	280.00	----	----

**Figure 6:**
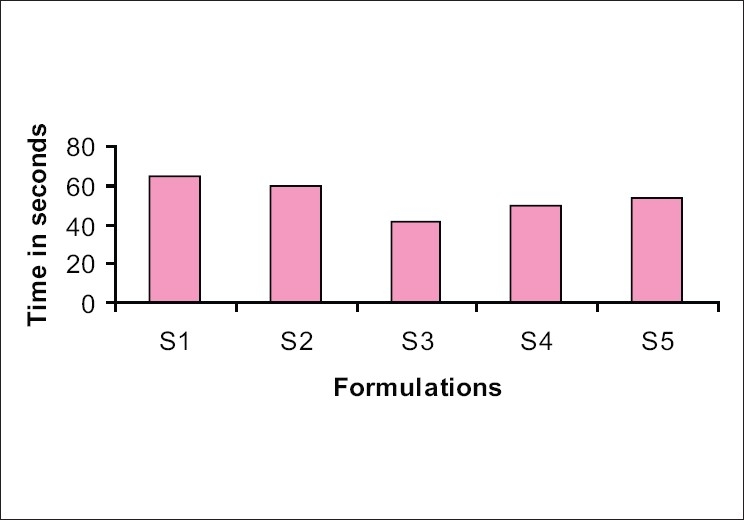
Disintegration profile of the selected batch

**Figure 7:**
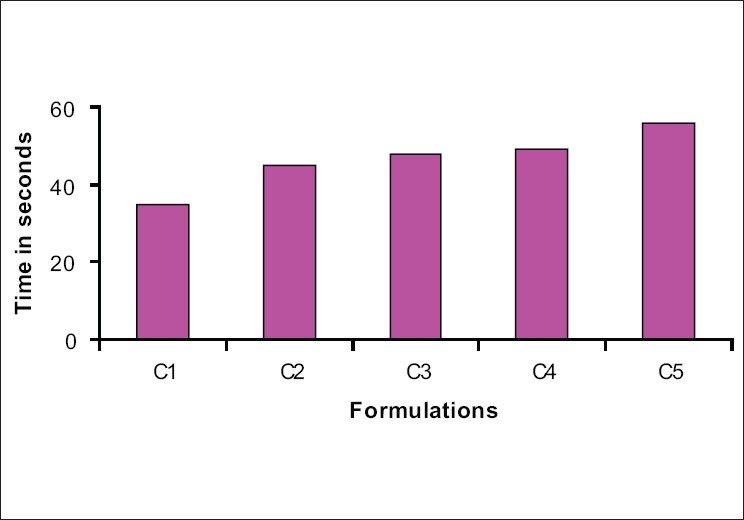
Disintegration profile of the selected batch

DT stands satisfactory with the time limit of <1 min for all the batches, as shown in Figures 6 and 7. Now, the question exists for fixing the best batch among the rest. Hence, for the comparative study, one with the same percentage was selected (2%). The batches S_3_ and C_1_ were selected to carry out further evaluation [Table 5].

**Table 5:** Evaluation parameters of the best formulations

Tablet evaluation parameters	Formula S_3_	Formula C_1_
Diameter	0.96	0.95
Thickness	0.36	0.38
Hardness	3.0	3.5
Friability	0.751	0.725
Weight variation	pass	pass
Disintegration time (sec)	42	32
Dissolution rate (%)	94.56	96.37

Evaluation of tableting properties of the selected batches confirm the standards prescribed in Indian pharmacopoeia. The results are given in Tables 6 and 7.

**Table 6:** Evaluation of tabletting parameters

Tablet parameters	Formula S_1_	Formula S_2_	Formula S_3_	Formula S_4_	Formula S_5_
Thickness (cm)	0.37	0.37	0.36	0.38	0.36
Hardness (kg/cm^2^)	2.6	2.8	3.0	2.6	2.6
Friability (%)	0.750	0.751	0.526	0.640	0.575

**Table 7:** Evaluation of tabletting parameters

Tablet parameters	Formula C_1_	Formula C_2_	Formula C_3_	Formula C_4_	Formula C_5_
Thickness(cm)	0.38	0.37	0.37	0.36	0.37
Hardness (kg/cm^2^)	2.8	2.8	2.6	2.4	2.6
Friability (%)	0.538	0.751	0.752	0.638	0.575

Disintegration time was found to be within 1 min and the percentage drug release, which confirms the *in vitro* bioavailability, was found to be 94.56 and 96.37, respectively, for S_3_ and C_1_, which proves the credibility of the selected products. The results are given in Table 8.

**Table 8:** Disintegration profile of the formulations

Formula	S_1_	S_2_	S_3_	S_4_	S_5_	C_1_	C_2_	C_3_	C_4_	C_5_
Disintegration time in seconds	65	60	42	50	54	35	45	48	49	53

With reference to Table 5, a comparison of the different parameters like hardness, friability, disintegration and dissolution was carried out and superiority of the tablets formulated with crosscarmellose was established.

## CONCLUSION

The aim of the present project is formulation development, evaluation and comparative study of superdisintergrants in the Cefixime 50 mg oral disintegrating tablet.

With the proof of different evaluation parameters listed in Table 8, it was concluded that C_3_ (CCS) was the best formulation.

Comparative evaluation studies proved that crosscarmelose is superior to sodium starch glycolate.
